# Supplementary material for: Empirical methods for controlling false positives and estimating confidence in ChIP-Seq peaks
Source: BMC Bioinformatics. 2008 Dec 5;9:523. doi: 10.1186/1471-2105-9-523 (PMC2628906; doi:10.1186/1471-2105-9-523)
Supplement: Additional File 1 — A variety of html documents from the USeq web site detailing the available applications, their best usage, output file type descriptions, command line menus, etc. [file 1471-2105-9-523-S1.zip › USeqUserGuides/JavaDoc.zip!/JavaDoc/trans/main/package-frame.html]

trans.main


trans.main

Classes 
  
BindingPeak
  
BindingRegionGraphPrinter
  
ChromSet
  
ExportIntervalData
  
FDRVsQValues
  
FDRWindowConverter
  
FindSubBindingRegions
  
IntensityPrinter
  
Interval
  
IntervalComparator
  
IntervalFilter
  
IntervalGFFPrinter
  
IntervalGraphPrinter
  
IntervalMaker
  
IntervalReportPrinter
  
LoadChipSetIntervalOligoInfo
  
LoadIntervalOligoInfo
  
MakeAllWindows
  
MergeWindowArrays
  
MultiSGRPrinter
  
MultiWindowIntervalMaker
  
Oligo
  
OligoIntensityPrinter
  
OverlapCounter
  
PeakPicker
  
PosRegion
  
RandomizeLabels
  
ReplaceWindowScores
  
SamChromosomeData
  
ScanChip
  
ScanChromosomes
  
ScanChromosomesCNV
  
ScanGenes
  
ScoredGene
  
ScoreIntervals
  
SetNumberIntervalMaker
  
SplitWindowArraysByChromosome
  
SubWindow
  
T2
  
T2Parameter
  
TestScanChip
  
WilcoxonRankSumTest
  
WilcoxonSample
  
WilcoxonSamplePair
  
WilcoxonSignedRankTest
  
Window
  
WindowBlockMaker
  
WindowBlockMakerTwoColor
  
WindowComparator
